# A novel *BRCA2* splice variant identified in a young woman

**DOI:** 10.1002/mgg3.1513

**Published:** 2020-11-07

**Authors:** Arianna Nicolussi, Francesca Belardinilli, Laura Ottini, Marialaura Petroni, Carlo Capalbo, Giuseppe Giannini, Anna Coppa

**Affiliations:** ^1^ Department of Experimental Medicine University of Roma “La Sapienza” Roma Italy; ^2^ Department of Molecular Medicine University of Roma “La Sapienza” Roma Italy; ^3^ Istituto Pasteur‐Fondazione Cenci Bolognetti Roma Italy

**Keywords:** *BRCA2*, hereditary breast/ovarian cancer, splice variant, VUS

## Abstract

**Background:**

*BRCA1*/*2* VUSs represent an important clinical issue in risk assessment for the breast/ovarian cancer families (HBOC) families. Among them, some occurring within the intron‐exon boundary may lead to aberrant splicing process by altering or creating de novo splicing regulatory elements or unmasking cryptic splice site. Defining the impact of these potential splice variants at functional level is important to establish their pathogenic role.

**Methods:**

Genomic DNA was extracted from peripheral blood sample of a young woman affected with breast cancer belonging to a HBOC family and the entire coding regions of the *BRCA1* and *BRCA2* genes were amplified using the Ion AmpliSeq BRCA1 and BRCA2 Panel. The *BRCA2* c.682‐2delA variant has been characterized by RT‐PCR analysis performed on mRNA extracted from blood and lymphoblastoid cell line.

**Results:**

We demonstrated that a novel *BRCA2* c.682‐2delA variant at the highly conserved splice consensus site in intron 8 disrupts the canonical splice acceptor site generating a truncated protein as predicted by several bioinformatics tools. Segregations analysis in the family and LOH performed on proband breast cancer tissue further confirmed its classification as pathogenic variant.

**Conclusion:**

Combining different methodologies, we characterized this new *BRCA2* variant and provided findings of clinical utility for its classification as pathogenic variant.

## Graphical abstract

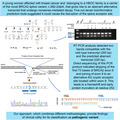

## 1 INTRODUCTION

*BRCA1* (OMIM#113705) and *BRCA2* (OMIM#600185) germline pathogenic variants are associated with an increased risk for breast and ovarian cancers, while a smaller increase in cancer risk is observed for stomach, uterine, cervix, and colon cancer for *BRCA1*, and prostate, pancreatic, gallbladder, stomach cancer, and melanoma for *BRCA2* (Palma et al., 2006). They are typically found in 25%–30% of the breast/ovarian cancer families (HBOC) subjected to genetic testing (Coppa et al., 2018; Economopoulou et al., 2015), representing a precious information for the clinical management of these patients. The recent advances in sequencing technologies applied to genetic diagnosis improved the speed and efficiency of DNA testing not only leading to the identification of many other genes involved in breast/ovarian cancer (Angeli et al., 2020), but also increasing the number of newly identified Variants of Uncertain Significance (VUSs). The great majority of pathogenic *BRCA* variants are protein‐truncating variants that may cause the complete loss of function of the mutant protein (Spurdle et al., 2012). Considering that *BRCA* genes are highly polymorphic, missense or synonymous variants are collectively common and only a small minority of non‐truncating *BRCA* variants are classified as pathogenic. Therefore, VUSs represent an important clinical issue in risk assessment for HBOC families (Richter et al., 2013).

Among VUSs, some occur within the intron‐exon boundary and may lead to aberrant splicing process by altering or creating de novo splicing regulatory elements, or unmasking cryptic splice sites, resulting in partial or total deletion of exon/s or retention of the adjacent intron (Thomassen et al., 2012). Defining the impact of these potential splice variants at the functional level is important to establish their pathogenic role. Here we describe a novel variant at the highly conserved splice consensus site in *BRCA2* intron 8, identified in a young woman affected with breast cancer and belonging to a HBOC family.

## 2 MATERIAL AND METHODS

### 2.1 Patients

The proband of HBOC Italian family (Caucasian), enrolled at the Hereditary Tumors section of the Policlinico Umberto I, University La Sapienza, was subjected to *BRCA1*/*2* mutation screening. Segregation analysis was performed on three relatives. A careful pretest counseling has been offered to proband and her relatives to obtain an informed consent. Proband's paraffin‐embedded tumoral tissue was obtained to perform loss of heterozygosity (LOH) analysis. All investigations were approved by Ethics Committee of the University of Roma “La Sapienza” (Prot.: 669/17) and conducted according to the principles outlined in the declaration of Helsinki.

### 2.2 *BRCA1*/*2* mutation screening

Genomic DNA was extracted from peripheral blood samples using a commercial kit (QIAamp Blood Kit, Qiagen, Valencia, CA). The entire coding regions of the *BRCA1* and *BRCA2* genes were amplified using the Ion AmpliSeq BRCA1 and BRCA2 Panel, the Ion AmpliSeq^TM^ Library Kit 2.0 and the Ion Xpress^TM^ Barcode Adapter 1‐16 Kit (Thermo Fisher Scientific, Waltham, MA, USA) to generate Ion Torrent adapter‐barcode ligated libraries, according to the manufacturer's procedures, as previously described (Nicolussi et al., 2019). The average depth of total coverage was set at minimum 500× and for variant calls at minimum of 100×. The NGS results were confirmed by direct sequencing using an ABI PRISM DyeDeoxy Terminator Cycle Sequencing Kit and an ABI 3130XL Genetic Analyzer (Applied Biosystems, Warrington, UK) as described previously (Coppa et al., 2014). Reference sequences were GenBank NM_007294.3 (NP_009225.1) and NM_000059.3 (NP_000050.3) for *BRCA1* and *BRCA2*, respectively. DNA variant nomenclature followed current guidelines of the Human Genome Variation Society (http://www.hgvs.org/rec.html).

### 2.3 Lymphomonocyte EBV‐immortalization, RNA extraction, cDNA synthesis, RT‐PCR, and identification of splicing aberrations

Lymphoblastoid cell line (LCL) was generated from proband using a standard protocol (Neitzel, 1986). To perform the amplification of natural and aberrant transcripts of the *BRCA2* gene, LCL was untreated and treated with cycloheximide (CHX, 100 μg/ml) and puromycin (400 μg/ml) for 4, 6, and 8 h in order to block NMD.

Total RNA extraction from LCL and from blood was performed using TRI Reagent® (Sigma‐Aldrich, Co.) and PAXgene Blood RNA Kit (Qiagen, Valencia, CA), respectively, according to the manufacturer's instructions. One microgram of the RNA was retro‐transcribed and PCR amplified as described (primer sequences are available on request) (Nicolussi et al., 2014).

### 2.4 Bioinformatic analysis of variant sequences

The following splicing prediction bioinformatic tools were used: MaxEntScan::score3ss (http://hollywood.mit.edu/burgelab/maxent/Xmaxentscan_scoreseq_acc.html), Mutation taster (http://www.mutationtaster.org/), NetGene2 Server (http://www.cbs.dtu.dk/services/NetGene2/), Human Splicing Finder 3.0 (http://www.umd.be/HSF3/), Alternative Splice Site Predictor (ASSP) (http://wangcomputing.com/assp/index.html), ESEfinder3.0 (http://rulai.cshl.edu/cgi‐bin/tools/ESE3/esefinder.cgi?process=home), Splice Site Prediction by Neural Network (NNSPLICE) (http://www.fruitfly.org/seq_tools/splice.html).

The meaning of the variants not yet reported in the literature and in the databases was established according to ENIGMA (https://enigmaconsortium.org/) and ACMG criteria (Richards et al., 2015).

### 2.5 Tissue processing and LOH

Hematoxylin/eosin stained histology slides were examined by a pathologist (I.P.) to identify areas of at least 70% breast carcinoma cells. FFPE derived genomic DNA was isolated from three 10‐*μ*m paraffin slides using standard protocols (Belardinilli et al., 2015) and LOH analysis has been performed by direct sequencing (primers are available on request).

## 3 RESULTS

### 3.1 The case

**FIGURE 1:**
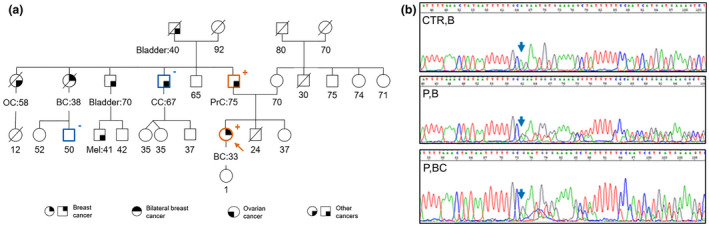
(a) Pedigree of HBOC family (proband is indicated with an arrow). Tested relatives are marked with “+” for mutation carriers and “−” for wild‐type. (b) Electropherograms of DNA from proband blood (P,B) and tumor tissue (P,BC) and from control (CTRL,B). Cancer type and age at diagnosis are reported and described as: BC, breast cancer; Bladder, bladder cancer; CC, colon cancer; OC, ovarian cancer; PrC, prostatic cancer; Mel, melanoma

In a 33‐year‐old Italian breast cancer patient belonging to a HBOC family (BRCAPro score: 88%) with no other known pathogenic *BRCA* mutation, we identified a novel c.682‐2delA variant involving the AG consensus at the 3′ end of *BRCA2* intron 8 (Figure 1a). Five out seven bioinformatics prediction tools suggested it could cause the disruption of the splice acceptor site, likely resulting in an aberrant protein product (Table 1).

**TABLE 1:** Selected splicing prediction bioinformatic tools

Bioinformatic prediction tool	Website	Prediction
MaxEntScan::score3ss	http://hollywood.mit.edu/burgelab/maxent/xmaxentscan_scoreseq_acc.html	Variation in the score of the splicing site: ttaaactataatttttgcagAAT (7.06) (wt)
		ttaaactataatttttgcgAAT (−6.91) (mu)
MutationTaster	http://www.mutationtaster.org/	Disease causing: alteration within used splice site likely to disturb normal splicing. Loss of an Acceptor Splice site and gain of two new potential acceptor sites (score 0.48, 0.38)
NetGene2 Server	http://www.cbs.dtu.dk/services/NetGene2/	Loss of an Acceptor Splice site
Human Splicing Finder 3.0	http://www.umd.be/HSF/HSF.shtml	Acceptor Splice site broken (−65.9%; wt:86.68 vs. mu:29.56)
		Acceptor Splice site gain (+66.84%; wt:39.33 vs. mu:65.62)
Alternative Splice Site Predictor (ASSP)	http://wangcomputing.com/assp/index.html	Loss of unclassified acceptor (score 6.618)
ESE finder	http://rulai.cshl.edu/cgi‐bin/tools/ESE3/esefinder.cgi	No changes predicted
Splice Site Prediction by Neural Network (NNSPLICE)	https://www.fruitfly.org/seq_tools/splice.html	No changes predicted

**FIGURE 2:**
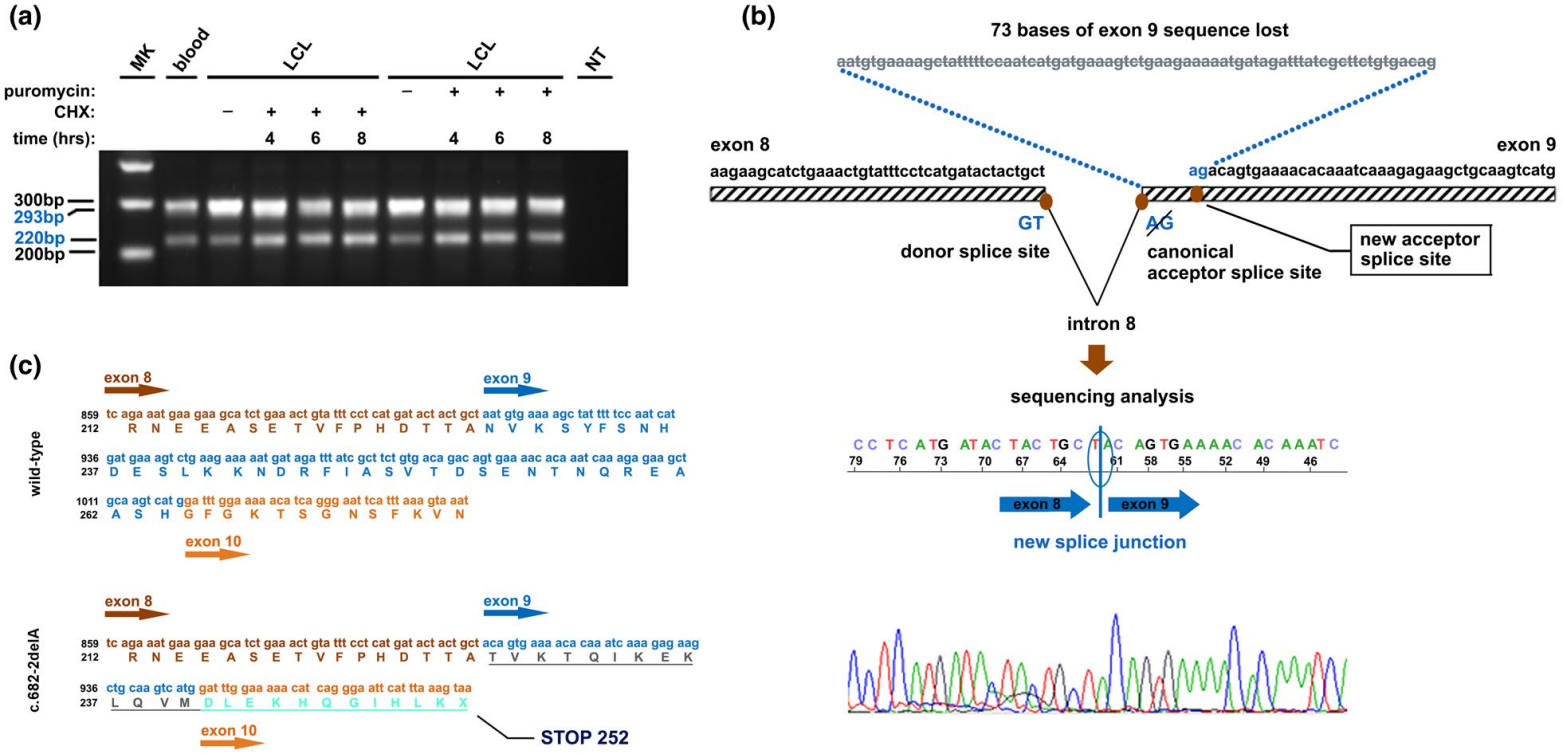
*BRCA2* c.682‐2delA variant identified in the proband of HBOC family. (a) PCR amplification of the alternative transcripts (lower band) in mRNA from blood and LCL exposed or non‐exposed to cycloheximide (CHX) or puromycin for 4, 6, and 8 h. (b) Schematic representation and electropherograms of the aberrant transcript lacking 73 bases of *BRCA2* exon 9. Aberrant transcript is generated by an alternative cryptic acceptor site within exon 9. (c) Partial sequence of the BRCA2 protein with the premature stop codon at residue 252 (p.Asn228Thrfs). LCL, lymphoblastoid cell line; MK, marker; NT, no template

The functional consequence of this mutation was ascertained by RT‐PCR and sequencing analysis of the transcripts which detected two band: one compatible with the wild type transcript (293 bp upper band) and the second compatible with predicted aberrant transcript (220 bp lower band) (Figure 2a). Direct sequencing of this PCR product indicated skipping of the first 73 bases of *BRCA2* exon 9 and joining of exon 8 to an alternative AG cryptic acceptor site located within exon 9 (Figure 2b). This leads to a frameshift and introduction of a premature stop codon at residue 252 (p.Asn228Thrfs) (Figure 2c). RT‐PCR analysis on cDNA from patient LCL indicates that cycloheximide (CHX, 100 μg/ml) or puromycin (400 μg/ml) stabilize the aberrant transcript suggesting it might be subjected to nonsense mediated decay (NMD). No other aberrant transcripts were detected in the *BRCA2* gene (Figure 2a).

The family pedigree showed that two aunts on the paternal side died with ovarian and breast cancer at the age of 58 and 38, respectively (Figure 1a). The segregation analysis performed in three relatives revealed that the variant cosegregates with the disease in one case of prostate cancer occurring in the father, but not in the relative affected with colon cancer at the age 67 (Figure 1a). Sequencing of the DNA from proband tumor tissue showed enrichment of the mutant allele possibly suggesting LOH (Figure 1b).

## 4 DISCUSSION

In this article, we describe a novel c.682‐2delA splice variant of *BRCA2*, identified in an early onset breast cancer proband belonging to a HBOC family. To establish the significance of novel variants that may alter the splicing process, the Evidence‐based Network for the Interpretation of Germline Mutant Alleles (ENIGMA) consortium Splicing Working Group propose a multifactorial likelihood analysis approach (Spurdle et al., 2012) based on the use of a variety of prediction bioinformatics tools combined to *in vitro* analysis of mRNA from biological material of a variant carrier to confirm the predictions. Segregation analyses and/or report of family history further contributes to establish the posterior probability of pathogenicity (Thomassen et al., 2012). We applied this integrated approach to ascertain the significance the *BRCA2* c.682‐2delA variant. Multiple prediction tools foresaw a very high possibility it disrupts the canonical splice acceptor site. Our data on mRNA extracted from patient's blood or LCL clearly confirmed that this variant gives rise to an aberrant alternative transcript that undergo nonsense‐mediated decay. In addition, the aberrant transcript codes for a truncated protein with loss of all functional domains at the C‐terminal. A similar outcome was previously reported for the c.682‐2A>C variant, described as class V in ClinVar database (https://www.ncbi.nlm.nih.gov/clinvar/). Similarly to our variant, it also leads to loss of the first 73 bp of exon 9 and the generation of a truncated protein (p.Asn228ThrfsTer25) (Santos et al., 2014). On these bases we proposed that the novel c.682‐2delA *BRCA2* variant should be considered a class V pathogenic variant (Richards et al., 2015). The high number of cancers observed in this family, such as two cases of early onset breast cancer and an ovarian cancer case, strongly suggest this is a cancer predisposing variant. Consistently, the variant segregates with a prostate cancer case, sustaining the observation that *BRCA2* variants are predominantly associated with increased risks of breast and prostate cancers (Lecarpentier et al., 2017) and less with colon cancer.

The enrichment of the mutant allele observed in the proband cancer tissue suggest LOH, although a contamination with adjacent normal tissue during the microdissection cannot be excluded. On the other hand, it is possible that LOH in *BRCA* mutation carriers is not necessarily an early event during the tumorigenesis and the haploinsufficiency could represent another valid alternative pathogenetic mechanism (Santos et al., 2014). Several studies *in vivo* (King et al., 2007) and *in vitro* (Pathania et al., 2014) demonstrated that the haploinsufficiency contributes to the *BRCA*‐related breast tumorigenesis determining multiple defects of stalled fork repair and increasing the replication stress and DNA breaks. Moreover LOH is often a late and random event that affects only a fraction of the cancer cells (King et al., 2007) and its determination may be a clinically useful biomarker to predict resistance to DNA damaging agents in patients with hereditary breast/ovarian cancer (Maxwell et al., 2017).

In conclusion, in this work we described a novel *BRCA2* splice variant identified in a 33‐year‐old breast cancer patient belonging to a HBOC family. Our approach, which combines different methodologies, has been able to characterize this new *BRCA2* variant and provide findings of clinical utility for its classification as pathogenic variant.

## CONFLICT OF INTEREST

Authors declare no conflicts of interest.

## AUTHOR'S CONTRIBUTIONS

Arianna Nicolussi and Anna Coppa contributed to conception and design and drafting the manuscript. Arianna Nicolussi, Francesca Belardinilli, Carlo Capalbo, and Marialaura Petroni contributed to analysis and interpretation. Laura Ottini and Giuseppe Giannini contributed to critical revision of the manuscript.
